# Development of a methodology to measure the effect of ergot alkaloids on forestomach motility using real-time wireless telemetry

**DOI:** 10.3389/fchem.2014.00090

**Published:** 2014-10-13

**Authors:** Amanda M. Egert, James L. Klotz, Kyle R. McLeod, David L. Harmon

**Affiliations:** ^1^Ruminant Nutrition Laboratory, Department of Animal & Food Sciences, University of KentuckyLexington, KY, USA; ^2^Forage-Animal Production Research Unit, Agricultural Research Service, United States Department of AgricultureLexington, KY, USA

**Keywords:** forestomach, contractions, motility, rumen pressure, telemetry, ergot alkaloids, tall fescue

## Abstract

The objectives of these experiments were to characterize rumen motility patterns of cattle fed once daily using a real-time wireless telemetry system, determine when to measure rumen motility with this system, and determine the effect of ruminal dosing of ergot alkaloids on rumen motility. Ruminally cannulated Holstein steers (*n* = 8) were fed a basal diet of alfalfa cubes once daily. Rumen motility was measured by monitoring real-time pressure changes within the rumen using wireless telemetry and pressure transducers. Experiment 1 consisted of three 24-h rumen pressure collections beginning immediately after feeding. Data were recorded, stored, and analyzed using iox2 software and the rhythmic analyzer. All motility variables differed (*P* < 0.01) between hours and thirds (8-h periods) of the day. There were no differences between days for most variables. The variance of the second 8-h period of the day was less than (*P* < 0.01) the first for area and less than the third for amplitude, frequency, duration, and area (*P* < 0.05). These data demonstrated that the second 8-h period of the day was the least variable for many measures of motility and would provide the best opportunity for testing differences in motility due to treatments. In Experiment 2, the steers (*n* = 8) were pair-fed the basal diet of Experiment 1 and dosed with endophyte-free (E−) or endophyte-infected (E+; 0 or 10 μg ergovaline + ergovalinine/kg BW; respectively) tall fescue seed before feeding for 15 d. Rumen motility was measured for 8 h beginning 8 h after feeding for the first 14 d of seed dosing. Blood samples were taken on d 1, 7, and 15, and rumen content samples were taken on d 15. Baseline (*P* = 0.06) and peak (*P* = 0.04) pressure were lower for E+ steers. Water intake tended (*P* = 0.10) to be less for E+ steers the first 8 h period after feeding. The E+ seed treatment at this dosage under thermoneutral conditions did not significantly affect rumen motility, ruminal fill, or dry matter of rumen contents.

## Introduction

Numerous factors affect motility of the reticulorumen including diet composition, feed and water intake, environmental temperature, feeding vs. resting activity, volatile fatty acid concentrations, and metabolic conditions, such as hypocalcemia, as well as individual animal variation (Church, 1976). Additionally, many methods have previously been used for measuring forestomach motility, such as electromyography (McLeay and Smith, 2006; Poole et al., 2009), radiotelemetry (Cook et al., 1986), and pressure-sensitive recordings of ruminal gas (Colvin and Daniels, 1965) or fluids (Dado and Allen, 1993). In order to adequately evaluate the effect of a treatment on rumen motility, one must first understand typical rumen motility patterns throughout the entire feeding cycle.

Ergot alkaloids, which are produced by a symbiotic endophyte associated with tall fescue grass (Lyons et al., 1986), cause fescue toxicosis in grazing livestock (Strickland et al., 2011). Fescue toxicosis syndrome can be costly for livestock producers due to decreased average daily gains, feed intake, milk production, and conception rates (Strickland et al., 2011). Westendorf et al. (1993) found that about 93–96% of ergot alkaloids consumed are absorbed or transformed in the gastrointestinal tract. Additionally, it was determined that only 50–60% of the ergot alkaloids administered in the diet are recovered in the abomasum, which means that a large portion (40–50%) of ergot alkaloids in the diet are metabolized or absorbed in the forestomach.

Recent research with ergot alkaloids has suggested that rumen motility may also be altered with endophyte-infected tall fescue consumption. For example, Foote et al. (2013) demonstrated that the DM percentage and dry contents of the rumen on a BW basis were greater for cattle that were ruminally dosed with endophyte-infected (**E+**) tall fescue seed compared to cattle dosed with endophyte-free (**E−**) seed. This finding could indicate a difference in particulate or liquid passage rates. One hypothesis is that reduced passage rate in E+ steers could be a result of decreased rumen motility. Ergot alkaloids, specifically ergotamine and ergovaline, have been shown to decrease contractions and increase baseline tonus of reticulorumen smooth muscle in sheep when administered intravenously (McLeay and Smith, 2006; Poole et al., 2009). Yet, there has not been research investigating the effect of ergot alkaloids or endophyte-infected tall fescue seed on rumen motility patterns in cattle. Furthermore, data on ruminal or oral dosing of ergot alkaloids and the effect on rumen motility is lacking.

Therefore, the objectives of Experiment 1 were to characterize rumen motility patterns relative to feeding using a pressure transducer and real-time, wireless telemetry system and determine when, relative to feeding, to measure motility. Using the time period as determined in Experiment 1, the objective of Experiment 2 was to investigate the effects of ruminal dosing of endophyte-infected tall fescue seed on rumen motility, rumen dry matter contents, and ruminal fill in cattle.

## Materials and methods

The procedures used in this study were approved by the University of Kentucky Institutional Animal Care and Use Committee. Experiments were conducted at the University of Kentucky C. Oran Little Research Center in Woodford County.

### Experiment 1

#### Animal management

Eight ruminally cannulated Holstein steers (*BW* = 321 ± 11 kg) were fed alfalfa cubes (% composition on a DM basis: *CP* = 16.8; *ADF* = 33.5; *NDF* = 43.1; *NFC* = 29.1; *TDN* = 59; *NE*_m_ = 5.09 MJ/kg) at 1.5 × NE_m_ once daily (0830 h) top-dressed with a trace mineral pre-mix (Kentucky Nutrition Service, Lawrenceburg, KY, USA; *NaCl* = 92–96%; *Fe* = 9275 ppm; *Zn* = 5500 ppm; *Mn* = 4790 ppm; *Cu* = 1835 ppm; *I* = 115 ppm; *Se* = 18 ppm; *Co* = 65 ppm) to meet or exceed nutrient requirements (NRC, 2000). Steers were housed indoors at 22°C in individual 3 × 3 m stalls and given *ad libitum* access to water.

#### Telemetry system

A wireless telemetry system (emkaPACK*4G* telemetry system, emka TECHNOLOGIES USA, Falls Church, Virginia) was used to monitor real-time pressure changes in the rumen. The system consisted of 2 receivers, 8 transmitters, and 8 bptVAP modules (pressure transducers). Wireless receivers were mounted securely to the wall outside of the pens in the room with the steers. The receivers were hardwired to a POE+ switch (8-port gigabit GREENnet POE+ switch, TRENDnet, Torrance, CA) that was connected to a laptop. All cable connections were made using E5 ethernet cables. Transducers were connected to their corresponding transmitters using the auxiliary port. During experimentation, the transducer and transmitter were housed in a 1 L cylindrical plastic container with screw-on lid (Gordon Food Service, Wyoming, MI), which replaced the cap in the cannula opening. A stainless steel female luer lock bulkhead adapter inserted into the bottom of the container served as the connection between the transducer and catheter. A female luer lock to 2.4 mm barb adapter connected the transducer to a 5.5 cm piece of silicone tubing (i.d. = 2.4 mm; o.d. = 4.0 mm) attached to the barb of the bulkhead adapter. The transducer was taped to the side of the container to prevent kinks in the tubing.

The catheter was a section of 96.5 cm long Tygon tubing (i.d. = 3.2 mm; o.d. = 6.4 mm) with 8 fused Tygon tubing cuffs and a 22.9 cm latex balloon (Bargain Balloons, Niagara Falls, NY) on one end, which was prefilled with 1 L of water. The cuffs enabled consistent placement of the balloons on the end of the catheter. On the other end of the catheter, a 3-way stopcock was connected by means of a female luer lock to 3.2 mm barb adapter. The catheter was weighted with approximately 300 g anchored approximately 4 cm from the top of the balloon. Balloons, which were replaced before each data collection period, were secured to the catheter using latex castration bands (Ideal Instruments, Neogen Corporation, Lansing, MI) placed over the balloon tongue and clamped tightly onto the catheter by plastic hose clamps (acetyl copolymer; i.d. minimum = 11.4 mm; i.d. maximum = 13 mm; Cole-Palmer Instrument Co., Vernon Hills, IL). Cheesecloth was placed into the container to prevent excessive movement of the transmitter. Upon submerging the water-filled balloon in the ventral sac of the rumen, the attached container was inserted into the cannula opening. A piece of nylon webbing over the lid of the container was secured to the cannula with nylon screws and thumb nuts (1/4–20; Non-Ferrous Fastener Inc., Chino, CA) to keep the container in place.

#### Signal calibration

Each transmitter and pressure transducer combination was manually calibrated using a sphygmomanometer connected to the “out” port of the transducer. Calibration was performed in the two-points (sampled) mode (20 and 200 mm Hg) of iox2 software (iox 2.9.4.27, emka TECHNOLOGIES USA) before data collection commenced on each day. To verify that calibration was successful, various amounts of pressure were applied with the pressure gage to check that values on the gage matched values in iox2 software (emka TECHNOLOGIES USA).

#### Data collection and analysis

Data were collected for 24 consecutive hours to capture the entire feeding cycle on three separate instances for each steer. Collection periods began, immediately following feeding, at 0830 and ended at 0830 the following day. Data were recorded and stored using iox2 software (emka TECHNOLOGIES USA) with a sampling rate of 100 pressure readings per second. Smoothing was set to 20 samples (200 ms) to help eliminate some background and movement noise in the signal.

The rhythmic analyzer in iox2 was used simultaneously while data were collected to analyze the raw rumen pressure data, identify ruminal contractions, and calculate the following parameters for each contraction: baseline, peak, amplitude, frequency, time to peak (TTP), relaxation time (RT), and area under the curve. The log and storage cadence was set to event related mode calculating the mean of these parameters for each event. By definition, for data to be considered an event or contraction, the signal must have increased at least 4 mm Hg from baseline (event threshold). Additionally, the contraction must have a slope value for the TTP start threshold of at least 0.500 mm Hg/s.

Individual water intake was also recorded using water meters every 8 h after feeding on collection days.

#### Statistical analysis

Statistical Analysis Systems software (SAS; SAS Inst. Inc., Cary, NC) was used to calculate duration (TTP + RT) of each contraction. Values for baseline, peak, amplitude, frequency, TTP, RT, duration, and area for each animal were averaged for each hour after data collection began using the Proc Means procedure of SAS 9.3. Hourly means for the above variables were analyzed using a Proc GLIMMIX with repeated measures model of SAS considering animal as a random effect and effect of hour and thirds (consecutive 8-h periods within d, i.e., h 1–8, 9–16, and 17–24) as fixed effects. Tests for differences between days for each variable were conducted using contrasts. The Proc TTEST of SAS was run to determine equality of variances between thirds of the day after feeding for amplitude, frequency, duration, and area.

### Experiment 2

#### Animals and treatments

The same eight ruminally-cannulated Holstein steers (*BW* = 378 ± 12 kg) used in Experiment 1 were paired by weight in a randomized complete block design. Within block, one steer was assigned to each treatment: E− (“KY 32”; 0 mg ergovaline + ergovalinine/kg DM) or E+ (“KY 31”; 2.87 mg ergovaline + ergovalinine/kg DM; 0.65 mg ergotamine + ergotaminine/kg DM) tall fescue seed treatment. Tall fescue seed was analyzed for ergovaline isomer and ergotamine isomer concentrations using a HPLC with fluorescence detection procedure modified from Yates and Powell (1988). Steers were pair-fed the basal diet in Experiment 1 once daily (0800 h) starting at 1.5 × NE_m_ (NRC, 2000). Thus, the E− steers only received the quantity of feed their paired E+ steer consumed the previous day. Tall fescue seed was ground by a grinder mixer (MX125, Gehl, West Bend, WI) to pass through a 3-mm screen. Immediately before feeding, all steers were dosed with 1.45 kg tall fescue seed through the cannula opening for 15 d. The E+ steers received 10 μg ergovaline + ergovalinine/kg BW. Therefore, a combination of E+ and E− seed was used to achieve this dosage level for the E+ treatment animals. Steers were housed indoors at the University of Kentucky C. Oran Little Research Center in Woodford County at 22°C in individual 3 × 3 m stalls. *Ad libitum* access to water was provided.

#### Telemetry system and signal calibration

Experiment 2 utilized the wireless telemetry system (emka TECHNOLOGIES USA) and signal calibration procedure as described previously.

#### Data collection and analysis

During the first 14 d of ruminal seed dosing, an 8-h data collection period began 8 h after feeding (1600 h) each day. Data were recorded and stored using iox2 software with a sampling rate of 100 pressure readings per second with smoothing averaging every 20 readings (200 ms). Water intake was recorded using water flow meters immediately after feeding, before collection, and after collection (i.e. every 8 h). The rhythmic analyzer in iox2 was used as it was in Experiment 1 for data analysis.

#### Blood collection

On d 1, 7, and 15, blood was collected from the jugular vein immediately before seed dosing and feeding. Blood samples were allowed to clot for 24 h at 4°C and centrifuged at 1,500 × g for 25 min (4°C). Serum prolactin concentrations were analyzed by radioimmunoassay procedures of Bernard et al. (1993). The intraassay CV was 10.1% and the interassay CV was 7.4%.

#### Ruminal evacuation

Complete manual evacuation of the rumen contents was conducted 8 h after feeding on d 15 through the cannula. Ruminal fill was measured for each steer by weighing total rumen contents. Three replicate samples (approximately 100 g each) were taken from the ruminal contents of each steer for DM analysis. The remaining contents were placed back into the rumen immediately after sampling.

#### Statistical analysis

Calculations were as described for Experiment 1. Values for baseline pressure, peak pressure, amplitude, frequency, TTP, RT, duration, and area under the curve for each animal were averaged over the 8 h period every day using the Proc Means procedure of SAS. Motility variables were analyzed as a randomized block design (RBD) with repeated measures for the effects of seed, day, and the interaction of seed × day. Water intake, DM intake, and ruminal content measures were analyzed as an RBD for the effect of seed. Serum prolactin was analyzed as a randomized block design with repeated measures for fixed effects of seed, day, and the interaction. Probability of Type I error less than 0.05 was considered significant.

## Results

### Experiment 1

Mean (± s.e.m.) water intakes for the first (1–8 h), second (9–16 h), and third (17–24 h) 8-h periods of the day were 30.28 ± 2.00, 6.14 ± 0.72, and 0.18 ± 0.04 L, respectively. Table 1 displays the mean value for each rumen contraction variable measured and the range between animals.

**Table 1:** **Mean values and range between animals for rumen contraction variables**.

**Item, units**	**Mean^a^**	**s.e.m.^b^**	**Range^c^**
Baseline, mmHg	22.98	2.68	7.28
Peak, mmHg	30.28	2.78	7.29
Amplitude, mmHg	7.29	0.46	1.07
Frequency, contractions/min	2.87	0.23	0.92
Time to peak, s	4.06	0.42	0.85
Relaxation time, s	5.23	0.50	1.04
Duration, s	9.29	0.73	1.34
Area, mmHg^*^s	30.47	2.99	6.93

a *Mean = overall mean, n = 576*.

b *s.e.m. = standard error of the mean, n = 8*.

c *Range = range of means among the 8 steers*.

**Figure 1:**
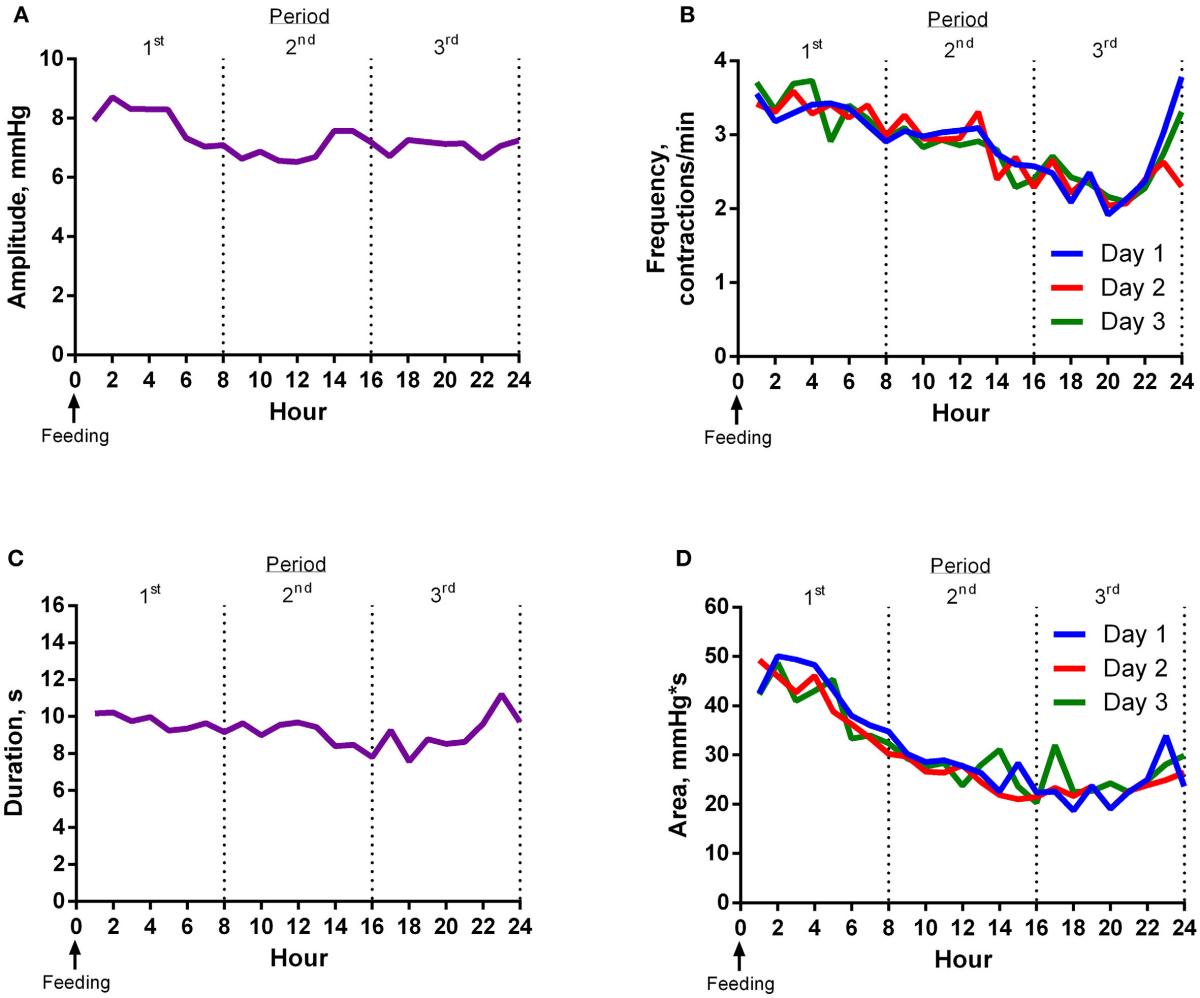
**Experiment 1 Results. (A)** Mean contraction amplitude of steers (*n* = 8) for each hour relative to feeding. The mean contraction amplitude of the first 8 h period was higher (*P* < 0.01) than the second. The mean contraction amplitude of the second 8 h period was not different (*P* = 0.96) from the third. **(B)** Mean contraction frequency of steers (*n* = 8) for each hour relative to feeding. Mean contraction frequency of day 2 was lower (*P* = 0.03) than day 3. The mean contraction frequency of the first 8 h period was higher (*P* < 0.01) than the second. The mean contraction frequency of the second 8 h period was different (*P* < 0.01) from the third. Additionally, the average of the first and third 8 h periods was not different (*P* = 0.35) from the second. **(C)** Mean contraction duration of steers (*n* = 8) for each hour relative to feeding. The mean contraction duration of the first 8 h period was longer (*P* < 0.01) than the second. The mean contraction duration of the second 8 h period was not different (*P* = 0.34) from the third. **(D)** Mean contraction area of steers (*n* = 8) for each hour relative to feeding. Mean contraction area of day 1 was lower (*P* = 0.03) than day 3. The mean contraction area of the first 8 h period was greater (*P* < 0.01) than the second. The mean contraction area of the second 8 h period tended (*P* = 0.07) to be greater than the third.

Contraction amplitude was greatest around feeding time (Figure 1A). Frequency of ruminal contractions was greatest at feeding time and decreased thereafter until 22 h after feeding (Figure 1B). The duration of contractions gradually decreased and then increased slightly 2 h before the next feeding (Figure 1C). Area under the curve for contractions decreased as time from feeding increased (Figure 1D).

All motility variables differed (*P* < 0.01) by hour and period (divided into 3, 8-h periods) of the day. The effect of day was not significant for most variables. However, the mean frequency of day 3 was higher (*P* = 0.03) than day 2, and mean area of day 3 was greater (*P* = 0.03) than day 1. Variance of the second 8 h period of the day was less than (*P* < 0.01) the first and third for area and less than (*P* < 0.05) the third for amplitude, frequency, and duration.

### Experiment 2

**Figure 2:**
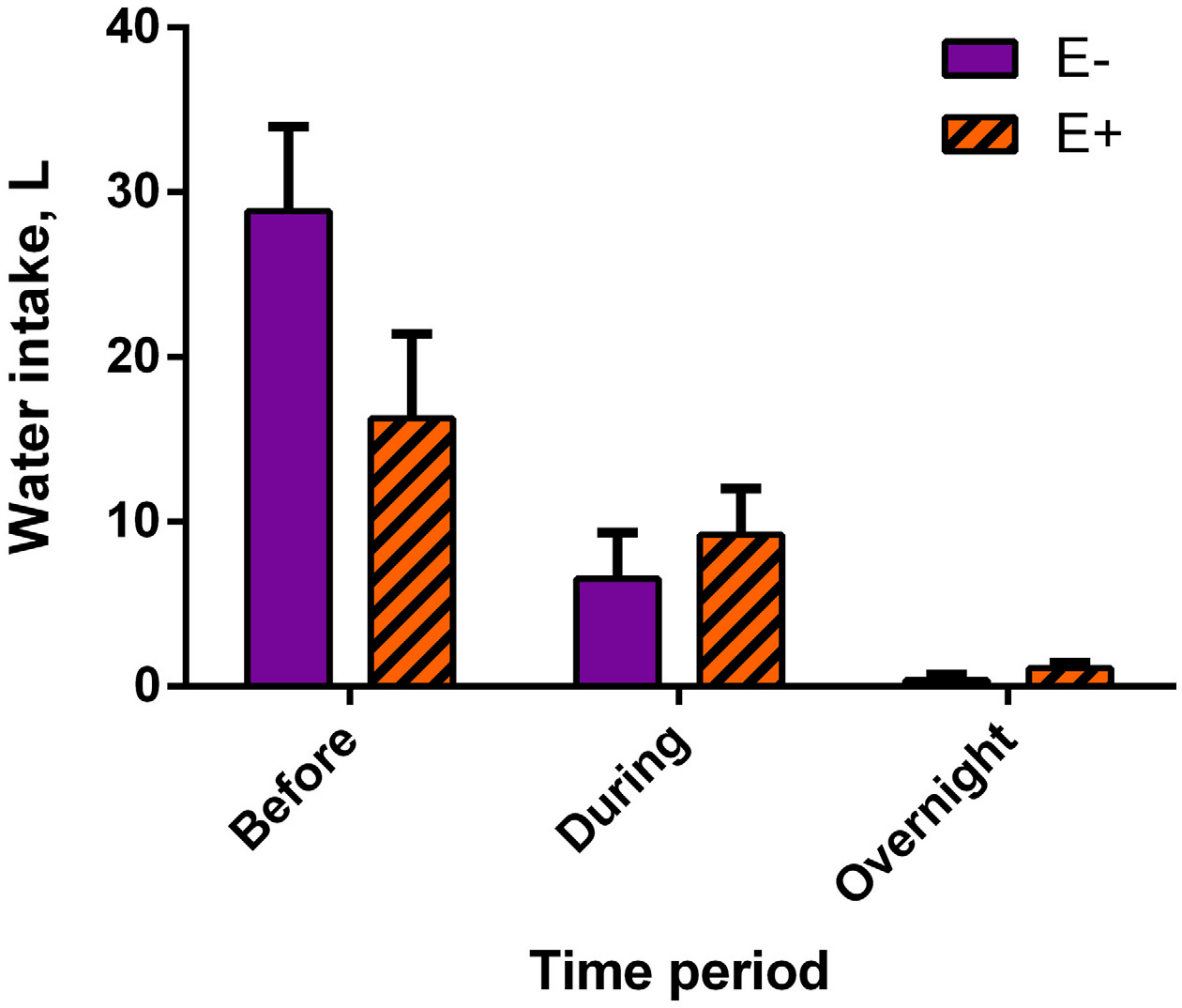
**Water intake by time period, relative to data collection, for endophyte-free (E−) and endophyte-infected (E+) tall fescue seed treated steers**. Steers in the E+ treatment group received 10 μg ergovaline + ergovalinine/kg BW daily. In the 8 h immediately before data collection commenced, water intake tended (*P* = 0.10) to be greater for E− steers. Water intake was not different between seed treatment groups during data collection (*P* = 0.55) or overnight (*P* = 0.23).

Mean water intake tended (*P* = 0.10) to be lower for E+ steers than for E− steers (16.27 ± 5.13 L and 28.86 ± 5.13 L, respectively) before data collection, meaning the 8-h period in between feeding and the start of data collection (Figure 2). There were no differences in water intakes between E− and E+ steers during data collection (E−: 6.54 ± 2.82 L; E+: 9.21 ± 2.82 L) or from the end of the data collection to feeding the next day (overnight; E−: 0.37 ± 0.35 L; E+: 1.10 ± 0.35 L).

**Figure 3:**
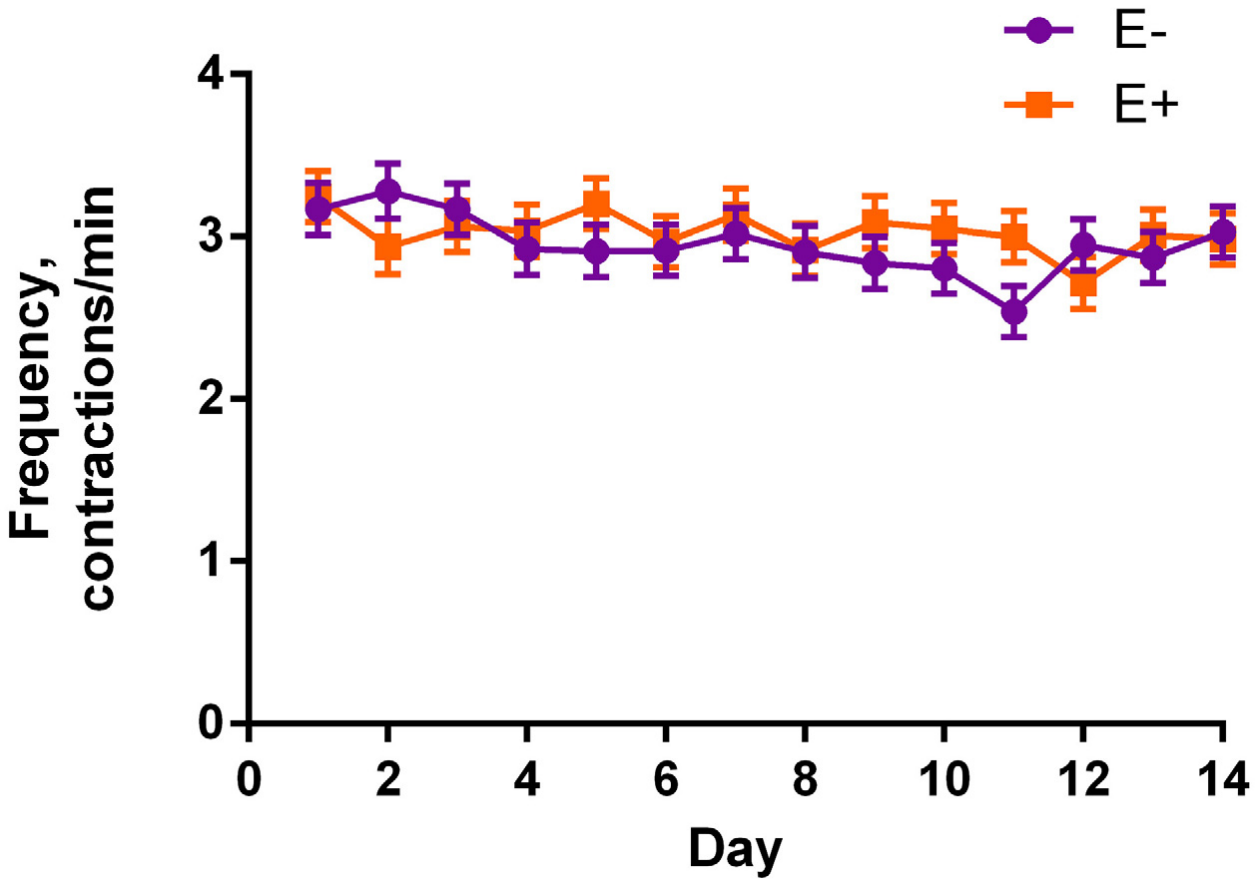
**Frequency of contractions for endophyte-free (E−) and endophyte-infected (E+) tall fescue seed treated steers each day of the experiment**. Steers in the E+ treatment group received 10 μg ergovaline + ergovalinine/kg BW daily. The effect of day was significant (*P* = 0.03), and there was a tendency (*P* = 0.10) for a seed × day interaction.

Table 2 shows the mean results of rumen motility variables between E− and E+ treated steers. Pressure at the peak of the contractions was smaller (*P* = 0.04) for E+ steers. There was also a tendency for baseline pressure to be smaller (*P* = 0.06) in E+ steers. The effect of day was significant (*P* < 0.05) for baseline pressure, peak pressure, and frequency, while tending (*P* = 0.10) to be different for duration. Contraction frequency had a tendency (*P* = 0.10) for a seed × day interaction (Figure 3).

**Table 2:** **Mean results for rumen motility contraction variables measured for 14 days in E− and E+ tall fescue seed treated steers**.

**Item**	**Seed treatment**		**s.e.m**.^c^	***P*-values**		
	**E−^a^**	**E+^b^**		**Seed**	**Day**	**Seed × Day**
Baseline, mm Hg	29.73	27.11	0.81	0.06	<0.01	0.43
Peak, mm Hg	36.68	34.30	0.65	0.04	<0.01	0.29
Amplitude, mm Hg	6.95	7.20	0.28	0.55	0.46	0.24
Frequency, contractions/min	2.95	3.02	0.12	0.68	0.03	0.10
Time to peak, s	4.29	4.43	0.11	0.43	0.35	0.39
Relaxation time, s	4.98	4.96	0.19	0.90	0.10	0.28
Duration, s	9.29	9.38	0.20	0.50	0.10	0.78
Area, mm Hg*s	28.86	31.55	1.91	0.36	0.13	0.84

a *E− = endophyte-free tall fescue seed*.

b *E+ = endophyte-infected tall fescue seed*.

c Standard error of the mean; n = 8

**Figure 4:**
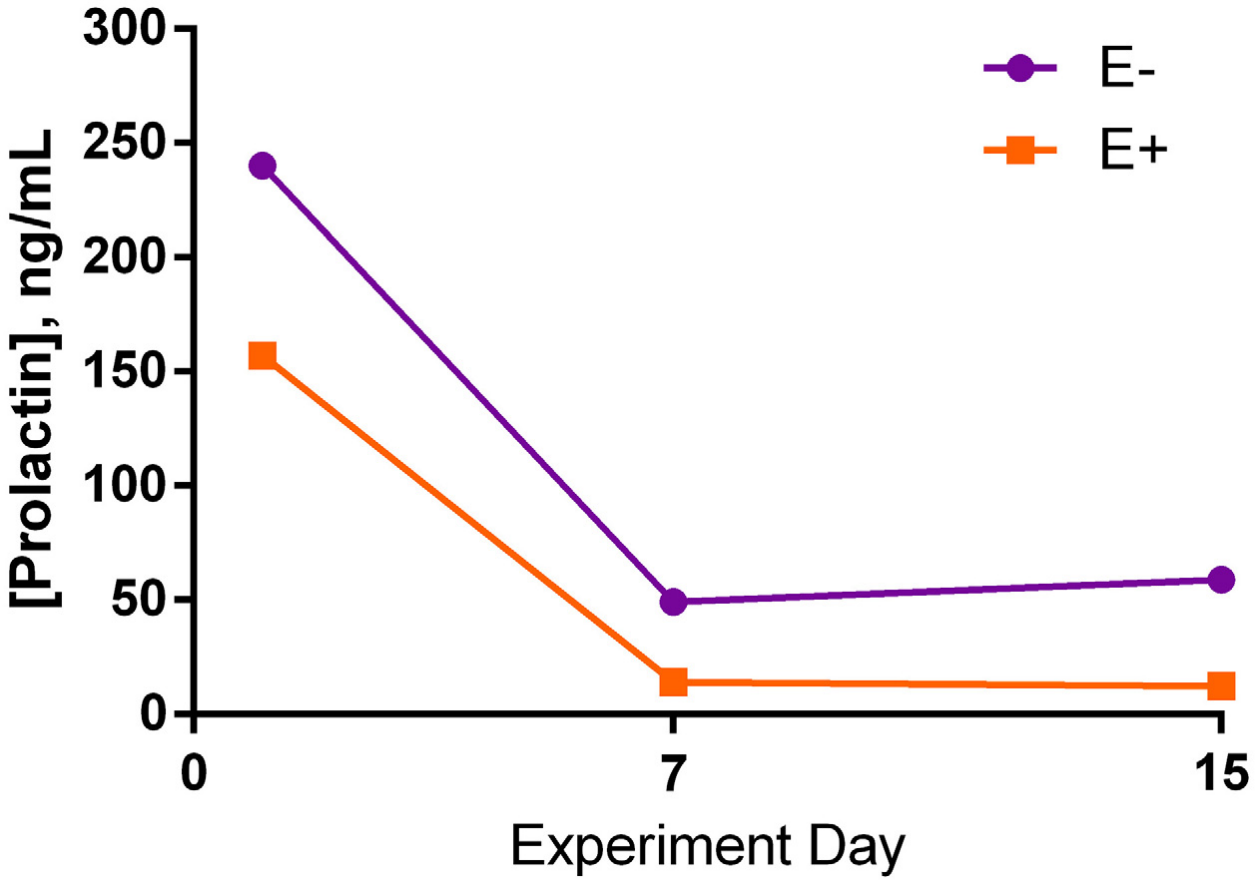
**Serum prolactin concentrations (*s.e.m*. = 50.4; *n* = 8) for endophyte-free (E−) and endophyte-infected (E+) seed-treated steers throughout the experiment**. Steers in the E+ treatment group received 10 μg ergovaline + ergovalinine/kg BW daily. The effects of seed, day, and the interaction were not significant (*P* > 0.05).

Serum prolactin was not different between seed treatments for any of the days (Figure 4). There was a large decrease in prolactin concentration between d 1 and d 7 of the trial for both treatment groups, although it was not significant. Comparison of d 7 and 15 showed relatively similar prolactin concentrations.

Table 3 displays the results of rumen evacuation and rumen content dry matter analysis. Particular consideration has been given to the relative DM intake around the time of evacuation between E− and E+ seed treated steers in an attempt to account for differences in rate of intake, despite the pair feeding situation. Dry matter intakes were not different (*P* = 0.71) between groups throughout the duration of the experiment due to the pair-feeding. Percent DM of ruminal contents, wet contents, and dry contents did not differ (*P* > 0.05) between E− and E+ steers. However, there was a tendency (*P* = 0.07) for the wet contents per 100 kg BW basis to be lower in E+ steers. For the 8 h immediately prior to evacuations on d 15, water consumption was not different (*P* = 0.13) between E− and E+ steers (27.89 ± 5.29 L and 16.66 ± 5.29 L, respectively.

**Table 3:** **DM intakes and ruminal contents measured by rumen evacuations on d 15 and DM analysis**.

**Item**	**Seed treatment**		**s.e.m**.^b^	***P*-value**
	**E−**	**E+**		
**INTAKES**				
DM^a^, kg	9.13	9.12	0.18	0.71
DM 8 h before evacuations, kg	9.84	7.59	1.02	0.22
DM 32 h before evacuations, kg	20.37	17.93	1.10	0.22
**RUMINAL CONTENTS**				
Percent DM	15.58	16.37	0.55	0.39
Wet contents, kg	66.50	59.68	3.47	0.16
Wet contents, kg/100 kg BW	17.58	15.80	0.47	0.07
Dry contents, kg	10.32	9.77	0.55	0.35
Dry contents, kg/100 kg BW	2.75	2.58	0.08	0.22
Dry contents, % of intake prior 8 h	105.27	143.59	20.46	0.25
Dry contents, % of intake prior 32 h	50.63	55.60	4.11	0.37

a *Mean DM intake for d 1 through d 14 with pair-feeding management*.

b Standard error of the mean; n = 8

## Discussion

### Experiment 1

This experiment was the first recorded adaptation of the emkaPACK*4G* wireless telemetry system for use in cattle. Most studies conducted with this system used canines (Bailey et al., 2011; McMahon et al., 2011) or non-human primates (Bruce et al., 2013). With the iox2 software, this system enables the measurement of many other variables beyond contraction amplitude and frequency, which are commonly the only variables reported as measurements of rumen motility (Attebery and Johnson, 1969; Bruce and Huber, 1973; Daniel, 1983; Cook et al., 1986). Additionally, other papers typically show values for these variables for the entire primary or secondary cycle (Froetschel et al., 1986; McSweeney et al., 1989; McLeay and Smith, 2006), whereas with this approach values for each contraction of the ventral sac are obtained. Because of this, it was difficult to find comparisons for some of these variables in published literature. The wireless aspect of this technology enables the animals to move freely and naturally in their environment. Moreover, the procedure for this technology is less invasive than other alternatives for measuring rumen motility, such as electromyography, and enables researchers to obtain a more detailed measurement of ruminal contractions.

Cannulation likely alters rumen motility and some measurements of motility may not be applicable to a non-cannulated animal. Mooney et al. (1971) found that cannulation decreased reticular contraction frequency during rest, but not during feeding or rumination. Also, amplitude of reticular contractions was significantly greater during feeding in intact animals. Frequency and amplitude of ruminal contractions within cannulated cattle also varies between sources. Attebery and Johnson (1969) reported frequencies between 1.74 and 2.23 contractions per min and amplitudes of 7.52–14.86 cm water in fed cows at various temperatures. However, observations were only taken for 30 min on 5 animals. Conversely, Daniel (1983) found an average frequency of the dorsal sac to be 1.13 ± 0.309 contractions per min and average amplitude of 14.7 ± 2.58 mm Hg prior to inducing hypocalcemia in cows. In this study, the mean frequency of ruminal contractions in steers was greater than those previously described, and the mean amplitude was generally lower. Differences could be attributable to the diet composition, method of measurement, time of recording relative to feeding, and length of recording.

Although statistical differences were found between certain days for frequency and area, graphically the days do not appear different. Therefore, these differences may not be physiologically relevant. Baseline and peak pressure displayed the greatest standard errors and ranges of all motility variables measured. This is likely due to the nature of the experiment and animal management as baseline and peak increase when the animal is laying down. Since the animals were allowed to move freely and stand up or lay down at will, the standard error of the mean and ranges for these parameters were more variable. Overall, small standard errors and ranges achieved here for measured parameters suggest that this approach to monitoring rumen motility is repeatable and consistent.

The second 8-h period of the day was the least variable for many measures of motility tested and had a moderate water intake. Therefore, it was concluded that measurements of motility for 9–16 h after feeding provide the best opportunity for testing differences in motility related to treatments because it provided the time when the pressure signal could be most consistently analyzed by the software due to less background noise. Feeding management will affect the values obtained and should be considered when designing experiments.

### Experiment 2

Research on the effects of endophyte-infected tall fescue or ergot alkaloids on rumen motility has been minimal. Previous research has been done via electromyography in sheep utilizing the direct intravenous injection of ergotamine and ergovaline (McLeay and Smith, 2006; Poole et al., 2009). However, there are no published data on the effects in cattle. Additionally, the route of administration could have an impact on the effects. Ergot alkaloids injected directly into the blood stream might cause a greater degree of biological reaction, such as vasoconstriction, than ergot alkaloids consumed orally or given intra-ruminally. As a result, this study attempted to delineate two aspects of information that are lacking: (1) the effect of ergot alkaloids on rumen motility specifically in cattle and (2) the effect on rumen motility when ergot alkaloids are dosed intraruminally (as opposed to intravenously).

Similarities between the ergoline ring of ergot alkaloids and dopamine enables ergot alkaloids to bind D2-dopamine receptors (Berde and Stürmer, 1978; Goldstein et al., 1980; Sibley and Creese, 1983). By binding to and activating these D2 receptors in the anterior pituitary gland, ergot alkaloids can inhibit the secretion of prolactin (Hurley et al., 1980; Schillo et al., 1988; Porter and Thompson, 1992) through second messenger responses (Larson et al., 1995). As a result, reduced serum prolactin concentrations have been used as an indicator of fescue toxicosis, yet do not indicate severity by level of decrease. There are multiple reports of cattle consuming endophyte-infected tall fescue or seed where a depression in serum prolactin concentration was shown (Schillo et al., 1988; Klotz et al., 2012; Koontz et al., 2012; Foote et al., 2013).

In this study, there was a large numerical decrease in serum prolactin concentrations from the first day of seed dosing to mid experiment in both seed treatment groups. The mean prolactin concentration for E+ steers was lower than E− steers throughout the experiment, yet, results were not statistically different. Therefore, prolactin data do not support that these steers were experiencing acute fescue toxicosis. Although it was chosen as an intermediate dosage from published studies showing reduced serum prolactin concentrations in E+ treated steers, the dosage rate of 10 μg ergovaline + ergovalinine/kg BW may have been too small to induce fescue toxicosis under thermoneutral conditions. Kim et al. (2013) administered approximately 8 μg ergovaline + ergovalinine/kg BW, whereas Foote et al. (2013) dosed 15 μg ergovaline + ergovalinine/kg BW. Both of these experiments successfully induced fescue toxicosis and utilized ground endophyte-infected tall fescue seed given intraruminally at thermoneutral temperatures, as was done in this study.

In contrast, other signs of fescue toxicosis were demonstrated. Reductions in DM intake were observed for many E+ steers, and E+ steers routinely consumed their daily ration at a slower rate than E− steers. Similarly, Koontz et al. (2012) showed a greater rate of dry matter intake at thermoneutral conditions for E− steers. Dry matter intake rate could not be controlled in this study with once daily feeding. This may help explain the baseline and peak pressure of E+ steers being lower than E− steers. For instance, if E− steers have consumed all of their feed, they would have likely been lying down more often during data collection, increasing the pressure, compared to the E+ steers, who still had food left to consume from their feed bunks. However, standing and laying behaviors were not monitored.

There was a tendency for a seed × day interaction for frequency of contractions (Figure 3). However, this is difficult to discern except that the E− steer contractions were less frequent than E+ steers on d 11.

There was also a tendency for greater water intake by E− steers compared to E+ steers during the first 8-h period following feeding, the period before motility data collection began. This was likely the result of the increased eating rate mentioned above and could have altered subsequent rumen motility (Church, 1976), although no significant differences were found. Aldrich et al. (1993) also reported water intake of steers was not changed with the consumption of tall fescue seed. Overall, the values obtained for rumen motility variables measured in this experiment agree with Experiment 1 and provide more support to the consistency of this approach.

Studies have demonstrated that cyclical contractions of reticuloruminal smooth muscle of sheep can be reduced or inhibited with the intravenous injection of ergot alkaloids (McLeay and Smith, 2006; Poole et al., 2009). This could potentially relate to a decreased passage rate of particulate or liquid matter, which could account for a reduction in intake as is commonly seen with ruminants experiencing fescue toxicosis. Unlike Foote et al. (2013), no differences were found in dry matter percentage of ruminal contents or dry contents (kg/100 kg BW) between E+ and E− steers in this study. The lack of effect on ruminal dry matter contents is likely a result of the E+ seed treatment not effectively inducing fescue toxicosis. Additionally, differences may be due to the time, relative to feeding, that rumen evacuations were conducted and rumen content samples were collected; Foote et al. (2013) gathered rumen content samples before feeding, whereas this study utilized rumen content samples collected 8 h after feeding.

Research has shown that duration of reticular contractions instead of frequency may have a larger influence on passage rate of ruminal fluid and particulate matter (Okine et al., 1989). The same theory could be applied to duration and frequency of ruminal contractions. However, seed treatment did not affect duration of contractions in this experiment.

## Conclusions

The emkaPACK*4G*wireless telemetry system can be used as an accurate, effective, and non-invasive tool to measure rumen motility and obtain detailed measurements of ruminal contractions in ruminally cannulated animals. Endophyte-infected tall fescue seed treatment at a dosage of 10 μg ergovaline + ergovalinine/kg BW under thermoneutral conditions for 14 days (which failed to induce acute fescue toxicosis) did not significantly alter rumen motility, ruminal fill, or dry matter of rumen contents. Therefore, it remains unclear as to whether ergot alkaloids or endophyte-infected tall fescue dosed intraruminally decreases rumen motility. Future experiments should focus on the interactions of ergot alkaloid dosage, ambient temperature, intake and feeding behavior, and rumen motility.

### Conflict of interest statement

The reviewer Lori L. Smith declares that, despite being affiliated with the same institution as authors Amanda M. Egert, Kyle R. McLeod and David L. Harmon, the review process was handled objectively and no conflict of interest exists. The authors declare that the research was conducted in the absence of any commercial or financial relationships that could be construed as a potential conflict of interest.
